# Migration-related Factors and Settlement Service Literacy: Findings from the Multi-site Migrants’ Settlement Study

**DOI:** 10.1007/s12134-023-01023-x

**Published:** 2023-03-20

**Authors:** Andre M. N. Renzaho, Michael Polonsky, Adnan Yusuf, Ahmed Ferdous, Michael Szafraniec, Bukola Salami, Julie Green

**Affiliations:** 1 Translational Health Research Institute, School of Medicine, Western Sydney University, Campbelltown, NSW 2560 Australia; 2 Maternal, Child and Adolescent Health Program, Burnet Institute, Melbourne, 3004 Australia; 3 Deakin Business School, Deakin University, 221 Burwood Hwy, Burwood, VIC 3125 Australia; 4 Multicultural NSW, 8/56 Station St E, Parramatta, NSW 2150 Australia; 5 Faculty of Nursing, University of Alberta, Edmonton, AB Canada; 6 Murdoch Children’s Research Institute, Parkville, VIC Australia; 7 School of Medicine, Western Sydney University, Penrith, NSW Australia; 8 Department of Paediatrics, University of Melbourne, Parkville, VIC Australia

**Keywords:** Settlement services literacy, Settlement services, Information, Literacy, Humanitarian migrants, Refugees

## Abstract

Migrants’ access and effective utilisation of settlement services depend on their level of settlement service literacy (SSL). However, SSL is multi-dimensional in nature and has many facets that are influenced by demographic and migration-related factors. Identifying factors that drive various components of SSL, and thus allowing for more focused development of specific dimensions, is critical. The aim of this study was to examine the relationship between components of SSL and migration-related and migrants’ demographic factors. Using a snowball sampling approach, trained multilingual research assistants collected data on 653 participants. Data were collected using face-to-face or online (phone and via video platforms such as Zoom and Skype) surveys. Our findings suggest that demographic and migration-related factors explained 32% of the variance in overall SSL; and 17%, 23%, 44%, 8%, 10% of the variance in knowledge, empowerment, competence, community influence, and political components of SSL respectively. SSL was positively associated with pre-migration and post-migration educational attainment, being employed in Australia, being a refugee, coming from the sub-Saharan region but negatively associated with age and coming from the East Asia and Pacific region. Across SSL dimensions, post-migration education was the only factor positively associated with the overall SSL and all SSL dimensions (except the political dimension). Employment status in Australia was also positively associated with competency and empowerment, but not other dimensions. Affiliating with a religion other than Christianity or Islam was negatively associated with knowledge and empowerment whilst being a refugee was positively associated with knowledge. Age was negatively associated with the empowerment and competency dimensions. The study provides evidence of the importance of some pre- and post-migration factors that can assist in developing targeted initiatives to enhance migrants’ SSL. Identifying factors that drive various components of SSL will allow for more focused development of specific dimensions and therefore is critical.

## Background

Organisation for Economic Co-operation and Development (OECD) countries are experiencing cultural pluralism as the result of increased migration. The number of international migrants has more than tripled since the 1970s, increasing from 84.5 million people in 1970 to 281 million people in 2020 (International Organisation for Migration, 2020). However, the number of migrants varies considerably across OECD countries, but overall one in 10 people within OECD countries are foreign-born (OECD, 2018, 2021). OECD countries are home to around 128 million international migrants, and this number has increased by 23% over the last decade alone (OECD, 2018). Some OECD countries such as the USA, Canada, Germany, the UK or Australia attract significant higher numbers of international migrants, with Australia ranked sixth among OECD countries for the intake of permanent migrants and second for the proportion of its population born overseas (29.9%) (OECD, 2021).

Host countries commonly provide a range of generalist and specialist settlement services to new migrants to assist them with their successful integration through participation in countries’ economy and society at large (OECD, 2018). In Australia, settlement services and programs for new migrants can be traced back to post World War II migration policies (Spinks, 2009), which have subsequently evolved in the last eight decades to meet needs arising from a migrant population growth and the resulting increasing cultural and linguistic diversity. In their current form, migrant settlement service programs are diverse, encompass the pre-migration Australian Cultural Orientation Program, general (e.g. government-funded mainstream services) and specialised settlement service tackling a broad range of social, cultural, economic, and human service needs (Commonwealth of Australia, 2021). The Humanitarian Settlement Program (HSP) is the largest such program and lasts 6 to 18 months, providing skill-based support and knowledge building during early stages of settlement to help migrants become self-reliant and active members of the community. It incorporates specialised and intensive services that focus on migrants with complex or high needs not met by other settlement services. The Settlement Engagement and Transition Support (SETS) program is a support mechanism that caters for migrants’ long-term needs after they leave the HSP. The SETS focuses on enhancing migrants’ social participation, personal and economic well-being, independence, and community connectedness. The SETS program provides tailored client services and community capacity building, but also refer clients to the Adult Migrant English Program (i.e. unlimited hours of English classes until migrants reach vocational English) and the Skills for Education and Employment program (e.g. to build migrants’ literacy, numeracy and basic computer skills) based on identified clients’ needs. (Commonwealth of Australia, 2021; Department of Home Affairs, 2020a). However, the SETS program is for those who have been in Australia less than 5 years.

## Literature Review: Effectiveness of Settlement Services

Globally, there have been numerous studies examining the coverage and effectiveness of settlement service programs for new arrivals (Dimitrov & Angelov, 2017, George, 2002, Shields et al., 2016, Varela et al., 2021). Across the research, there seems to be some confusion around three critical and inter-related concepts related to migrants’ settlement services within OECD countries—(1) migrant policies, (2) migrant settlement policies, and (3) migration policies (Shields et al., 2016). Strictly speaking, migrant policies are concerned with governmental administrative decisions that set out migration pathways and seek to minimise the likelihood the newcomers becoming a drain on the public purse. Such policies focus on newcomers’ likelihood of succeeding in their new host nation, including gaining employment and achieving economic independence (e.g. decisions that govern how overseas skills and qualifications are recognised), skills shortage, or vocational preparation (e.g. prioritising countries that speak host nation’s language).

In contrast, migrant settlement policies are concerned with government structures, systems and associated programmatic processes and legal frameworks that seek to address newcomers’ needs through their various phases of the migration journey. These include pre-arrival, on-arrival and integration (post-arrival) programs such as access to social and welfare services, health care, education and affordable accommodation. Migration policies, on the other hand, typically focus on protecting borders against illegal migration, security threats, and to regulate who enters or leaves the country (Shields et al., 2016; Shpaizman, 2010, Schmidt Sr, 2007).

Over the last four decades, most OECD countries have adopted migrant settlement policies that promote integration models, supporting cultural pluralism or ‘pluralist integration’. This approach allows newcomers to integrate into host cultures, but also supports them maintaining aspects of their home cultural identities, values and practices (Schmidt Sr, 2007). Such inclusive integration policies shape migrant settlement programs and often include both (a) formal and specialised programs and (b) generalised programs.

Formal programs focus on maximising employment and vocational outcomes, providing support for antecedents to effective integration, such as host language courses and overseas skill and qualification recognition. Formal programs also seek to maximise newcomers’ well-being by increasing access to host cultural societies, through adaptation programs including social safety net programs, affordable accommodation and health care. The emphasis is on autonomy and independence boosting activities such as the provision of driving lessons, food shopping tours, access to interpreting services and financial planning and banking lessons. Generalised programs, on the other hand, may include activities such as an induction to host cultural and socio-legal dimensions (e.g. programs promoting tolerance of racial and religious diversity, civic engagement and the host nation’s cultural values, (Schmidt Sr, 2007, Shields et al., 2016, Commonwealth of Australia, 2021).

Policy makers and service providers of migrant settlement services need to assess the effectiveness of the programs offered. However, assessing these services has been a challenge. Migrant settlement policies, programs and associated models of integration have evolved overtime and have been shaped by the political history of the host nations, the political orientation of governments and global forces. As such, new iterations of programs and policies are extensions of past initiatives, which may or may not have been effective. Additionally, evaluation and monitoring of the impact of settlement programs in OECD countries has focused on governmental fiscal frameworks and expenditure and associated short-term outcomes, rather than ensure new migrants and refugees can integrate and participate fully in their host country’s socio-economic, cultural and political system (OECD, 2017, Dimitrov & Angelov, 2017).

Most governments focus on the short-term financial consequences when revising migrant policies, rather than the longer term benefits (OECD, 2017, Dimitrov & Angelov, 2017). Failing to focus on the long-term impact of settlement services for migrants can lead to wrong conclusions and misaligned policies. For example, a study in Australia used a closed cohort model of the 2018–2019 permanent migrant intake to estimate and projected the lifetime fiscal impact of this intake over the next 120 years (until 2139). It found that, overall, the permanent migrant cohort was more fiscally positive than the existing Australian population, by an average of around $127,000 per person, reflecting an aggregate lifetime fiscal contribution of $20 billion greater than a comparably sized cohort of Australian-born individuals (Varela et al., 2021). The study also found that the average fiscal impact of permanent migrants was $139,000 more positive than that of a newborn from the Australian population. However, when assessing the costs of the humanitarian migrant program, it was reported that this costs $400,000 per person, with almost all of these costs being covered by the Commonwealth Government (Varela et al., 2021). Thus, while other countries face similar impacts (OECD, 2017) the long-term benefits of accepting migrants and providing them with adequate settlement services outweigh the short-term costs.

Another factor that may affect the assessment of migrant settlement service performance is the coverage of migrants’ social and health services, as social, welfare and healthcare systems take many forms across countries. This, in turn, means comparing a program with similar objectives is designed very differently and includes a diverse range of varying components. For example, in Denmark, Sweden, Canada, UK and Australia humanitarian migrants have free access to social and welfares services education, healthcare and receive allowances that allow them to have a reasonable quality of life (Correa-Velez et al., 2005; Jensen et al., 2011). Whereas in other countries such as the USA, national formal settlement policies have been variable, and individual States have differing programs as well, with the basic levels of services quite limited (Khullar & Chokshi, 2019; Kotovicz et al., 2018; Morris et al., 2009).

Within most countries, there is no or limited tracking and evaluation of migrant settlement services performance indicators. There are certainly no measures of migrants’ access and utilisation of such services. The complexity of service provision means that an evaluation tool needs to be developed that can cater for the heterogeneity of services and migrants themselves. Migrant-based factors as well as host country factors will impact both on the level of integration occurs and the types of supports that are needed. Therefore, migrants’ chances of successful integration are also a function of age at migration, similarity of identity, occupational and vocational demands, countries of origin, educational attainment including recognition of overseas skills and qualifications, the size of their communities and relatives in the country of destination, length of stay, competence in host nations’ language and personal suitability (George, 2002, Maksum, 2022; Carruthers, 2017).

As such, when evaluating services, governments need to also assess migrants’ ability to navigate and engage with these new unfamiliar, complex and multi-dimensional host environments and approaches to settlement services. Migrants must have adequate skills and knowledge to navigate and use effectively settlement services (basic level), the ability to critically evaluate settlement services available to them to ascertain their quality and identify solutions to address identified challenges or gaps (critical level) and the agency and social capital to activate changes in the course of their integration journey (political level) (Masinda, 2014). However, although the three settlement literacy levels (basic, critical, and political) have recently been empirically validated (Renzaho et al., 2022b), evidence on how these literacies are affected by demographic and migration-related factors is lacking (Barton, 1994; Barton & Hamilton, 2000; Luke & Freebody, 1999). Some of these factors have been used to evaluate various governments service utilisation of some settlement programs (Malmusi et al., 2010; Renzaho, 2007; Rodríguez-Izquierdo & Darmody, 2019). Findings from these studies suggest that any action on inequality in socio-economic determinants that affect migrant groups must not be deferred (Malmusi et al., 2010).

Therefore, the potential mismatch between migration policies and migrants’ settlement outcomes remains a concern and the situation can worsen if the host nation fails to recognise and effectively address settlement needs (Massey, 2020; Sassen, 2000). A settlement service literacy tool that accounts for political factors and migrants’ knowledge and competence, empowerment and community influence would provide more robust results allow for more effective decision-making to inform settlement service planning and delivery.

This research, therefore, seeks to understand how demographic factors influence migrants’ overall settlement service literacy. Given the multi-dimensional nature of SSL, we also examine whether variations in the dimensions of SSL can arise for different reasons, and thus be addressed in different ways. As such, the aim of this study was to examine how the validated dimensions of the SSL are impacted by individual demographic and migration-related factors. Identifying factors that drive various components of SSL will allow for more focused development of specific dimensions and therefore is critical.

## Methods

### Study Setting and Participants

The study was carried out in the states of New South Wales and Victoria, Australia; and received institutional ethics approval (HREC Approval Number: H13063). Briefly, the study focused on migrants who have been in Australian for 5 years or less and living in local government areas that are socio-economically disadvantaged (with an index of relative socio-economic disadvantage score of < 1000—a cut-off for socio-economic disadvantage)with the highest proportion of migrants (where 37–56% of the population were born overseas) (Australian Bureau of Statistics, 2016). To be representative, we selected a cross-section of migrant communities. The major language groups included in our study were Arabic, Burmese, Dari, Farsi and Tamil, coming from a range of communities with different countries of birth.

### Participants’ Recruitment

In each of our target local government area, the multilingual research assistants distributed translated leaflets and posters throughout their own networks, community associations, migrant service providers, religious institutions and community facilities. Participant recruitment was undertaken using translated participant information sheets, and written consent was obtained. Our target communities were difficult-to-access populations with significant barriers, culture shock and always on the move in search of affordable housing (De Maio et al., 2017; Renzaho et al., 2010, 2011). Therefore, we used a snowball sampling approach (Atkinson & Flint, 2001) which is valuable in targeting hard to research groups (Leighton et al., 2021), where multilingual research assistants recruited participants through their contacts, community associations, social media (Facebook), referrals from local migrant services and via ethno-specific and religious community groups (Leighton et al., 2021). Participants and others in turn referred their friends and/or family to the research team. Sampling was undertaken in local government areas with the highest concentration of the target migrant communities ensured inclusiveness and adequate coverage of the disadvantaged new migrant population, hence minimising potential bias and improve representativeness. We sought to have representative sample quotas and the community mobilisation continued until each quota was filled or each multilingual research assistant was unable to recruit further participants in their community group and local government area.

### Data Collection Procedures

Data were collected by 39 multilingual research assistants recruited from the target communities. (Shoultz et al., 2006) (17 in NSW and 22 in Victoria), who were trained and supervised by two project officers experienced in data collection monitoring and data quality assurance and control. Multilingual research assistants received three hours of training followed by a practice run of administering the materials, prior to data collection, to ensure they were familiar with the study instrument and instrument administration procedures. The training covered sampling techniques and how to minimise biased oversampling, ethical issues including confidentiality, duty of care towards respondents, and data quality and integrity, interview techniques, professional conduct, as well as COVID safe practices, in line with university requirements.

Data were obtained from 653 participants recruited between 7 August and 9 December 2020. The survey dates fell within three COVID-19 related events in both Victoria and NSW: before, during and after various COVID 19 lockdowns which restricted travel and social contact. Therefore, the survey instruments were administered using either face-to-face or online (phone and via video platforms such as Zoom and Skype) approaches. All face-to-face surveys were carried out in participants’ preferred language and preferred location including homes, public libraries, community organisations, public parks and cafes. Respondents were compensated with a AUD20 gift voucher for their time and effort, as the survey took over 90 min to complete.

### Measures

Table 1 summarises study variables and their transformation. The dependent variable was SSL, which was measured using a psychometrically tested SSL scale which was based on Masinda’s initial conceptual of SSL. The validated SSL scale has five dimensions with a total of 19 items scored using a five-point Likert scale (from 1 = strongly disagree to 5 = strongly agree). The five dimensions are knowledge (five items), empowerment (four items), competence (four items), community influence (four items) and political participation (two items). The psychometric properties supported a good model fit with strong construct validity and reliability (Renzaho et al., 2022b). The composite of each dimension was the summation of items divided by the number of items, rather than an index based on the item weights. The independent variables were migrants’ demographic factors and migration-related factors. Collected data included length of stay in Australia, age in years, marital status, sex, pre-migration educational attainment, post-migration educational attainment, employment status, pre-migration work experience, reason for migration and countries of birth. Demographic and migration-related variables provided multiple categorical options (Tables 1 and 2), but in order to increase cell sizes, some variables were collapsed as follows (0 as the reference): marital status: 1 = married/de facto vs. 0 = all other marital status, sex; 1 = males vs. 0 = females; employment in Australia: 1 = employed vs. 0 = unemployed; pre-migration work experience: 1 = worked prior to migration vs. 0 = did not work prior to migration; religion: 1 = Muslims, 2 = other religious affiliation vs. 0 = Christians; migration status: 1 = refugees vs. 0 = all other migration categories; and country of birth classified into four major groups based on World Bank regional classification:1 = sub-Saharan Africa, 2 = South Asia, 3 = East Asia and Pacific vs. 0 = Middle East and North Africa.

**Table 1:** Description of variables and transformations

Variable	Scale	Comments
SSL and SSL dimensions	Continuous	All SSL dimensions (knowledge, empowerment, competence, community influence, and political are average scores of their respective items measured on a scale of 1 to 5. SSL is measured as an average score of all SSL dimensions
Time in Australia	Continuous	Time in years and months (for example a value of 5.5 means 5 years and 6 months)
Age	Continuous	Age in years. No transformation required
Marital Status	Categorical	Married/de facto = 1, else 0
Gender	Categorical	1 = female, 2 = male
Pre-migration education	Ordinal	1 = never went to school in home country 2 = attended primary or secondary school in home country 3 = trade certificate, tertiary qualification, or university qualification in home country
Post-migration education	Ordinal	1 = never up-took education in Australia 2 = attended or attending English/primary or high school in Australia 3 = trade certificate, tertiary qualification, or university qualification, attending or completed in Australia
Employed	Categorical	0 = unemployed, 1 = employed
Pre-migration work	Categorical	0 = no, 1 = yes; [In response to the question: Before you came to Australia, did you do any paid work in a job, business or on a farm?
Region of origin	Categorical	Created following groups of ethnicities based on World Bank classification: Middle East & North Africa (MENA)—represented by Iraq, Iran, and Syria (reference group) Sub-Saharan South Asia—represented by Afghanistan, Bangladesh, India, and Nepal East Asia and Pacific (EAP)—represented by Myanmar
Religion	Categorical	Christian (reference group) Muslim All other religions
Refugee	Categorical	1 = yes, 0 = no

**Table 2:** Demographic characteristics of the sample

	*N*	Percent
	653	100
*Settlement service literacy (mean* ± *SD; scale: 1–5 points)*		
Total SSL	653	2.26 ± 0.64
Knowledge	653	2.88 ± 0.96
Empowerment	653	2.23 ± 0.97
Competency	653	3.53 ± 1.10
Community influence	653	2.98 ± 0.83
Political	653	1.55 ± 0.82
*Age in years (mean* ± *SD)*	653	37.00 ± 11.82
*Years in Australia (mean* ± *SD)*	653	2.64 ± 1.34
*Sex*		
Male	243	37.21
Female	409	62.63
Not revealed	1	0.20
*Pre-migration education*		
University or tertiary	250	38.28
Technical or trade certificate	49	7.50
Completed secondary school	140	21.44
Some secondary school	87	13.32
Completed primary school	39	5.97
Some primary school	37	5.67
Other education	13	1.99
Never attended school	38	5.82
*Marital status*		
Married	445	68.15
Single	156	23.89
Widowed	26	3.98
Divorced	10	1.53
Separated	10	1.53
De facto	6	0.92
*Living structure*		
With family	595	91.12
With friends	24	3.68
With family and friends	13	1.99
Alone	12	1.84
Alone but sometimes with others	1	0.15
No response	8	1.23
*Employment status*		
Self employed	4	0.61
Permanent/ongoing	104	15.93
Fixed term contract	26	3.98
Casual	69	10.57
Other	7	1.07
Unemployed	443	67.84
*Migration type*		
Refugees or humanitarian entrants	397	60.80
Family reunion	98	15.01
Education opportunities	56	8.58
Financial/economic	54	8.27
Political/asylum seeker	14	2.14
Other reasons	34	5.21
Region of origin		
Middle East and North Africa	288	44.1
South Asian region	221	33.8
Sub-Saharan African	82	12.6
East Asia and Pacific	62	9.5
Religion		
Christian	301	46.1
Muslim	221	33.8
Other	131	20.1

In cases where there were more than two categorical responses, we assigned one group as the comparison group. We then created dummy variables for the number of groups minus one (*n*-1). Thus, in the case of religion there were three classification—Christianity, Muslim and Other—we created two dummies reflective of whether a person was part of that group (1) or not (0), for Muslim and other. Thus, Christianity the largest religious group was the comparison group. In the case of region there were four regions—sub-Saharan African South Asian region, East Asia and Pacific and Middle East and North Africa. We created three dummies reflective of whether a person was part of that group (1) or not (0) for sub-Saharan African, South Asian region, East Asia region and Pacific region. Thus, Middle East and North Africa was the largest locational group, and was used as the comparison group.

### Statistical Analysis

We conducted ordinary least squares regression analysis to investigate the relationships between overall SSL and each of the five dimensions, along with migrants’ demographic and migration-related factors. As a first step, we analysed bivariate relationships by conducting bivariate regressions with overall SSL and of its five dimensions) as our dependent variable and one of the demographic and migration-related variables separately as the explanatory variable. We then conducted multiple regression analysis by including all demographic and migration-related factors as explanatory variables for SSL (or each of its five dimensions). For multiple regression analysis, we checked for the presence of multicollinearity using the variance inflation factor (VIF). Maximum VIF was 2.11 which is well below the cut-off value of 10 (Diamantopoulos & Winklhofer, 2001) or a stricter threshold of 3 recommended by Petter et al. (2007). Therefore, we concluded that multicollinearity did not appear to be a cause of concern for our analysis.

## Results

The demographic characteristics of participants are summarised in Table 2. Table 3 provides bivariate and multivariate standardized beta coefficients of the relationship between SSL and demographic and migration-related factors. In the bivariate analyses, the overall SSL was negatively associated with age, being married, coming from the East Asia and Pacific region, and being a refugee, but positively associated with being male, length of stay in Australia, pre- and post-migration educational attainment, being employed, pre-migration work experience and coming from sub-Saharan Africa. SSL was not associated with religion or South Asia as a region of origin. However, the impact of these variables on SSL sub-dimensions sometimes varied from the observed pattern for the overall SSL.

**Table 3:** Bivariate and multivariate OLS regression results (standardized estimates) (*N* = 653)

	Overall SSL		Knowledge		Empowerment		Competency		Community Influence		Political	
	Bivariate	*Multivariate*	Bivariate	*Multivariate*	Bivariate	*Multivariate*	Bivariate	*Multivariate*	Bivariate	*Multivariate*	Bivariate	*Multivariate*
Time in Australia	0.16***	*0.05*	0.06	*0.01*	0.12**	*0.03*	0.14***	*0.04*	0.12**	*0.04*	0.10*	0.09*
Age	− 0.20***	− 0.10***	− 0.07	* − 0.02*	− 0.18***	− 0.09*	− 0.29***	− 0.22***	− 0.04	*0.05*	− 0.02	*0.01*
Marital status (married/de facto)	− 0.08*	* − 0.01*	− 0.06	*0.01*	− 0.10**	* − 0.02*	− 0.03	*0.02*	− 0.06	* − 0.07*	− 0.02	*0.02*
Gender (male)	0.09*	*0.03*	0.02	* − 0.02*	0.07	*0.03*	0.14***	*0.06*	0.00	*0.00*	0.05	*0.02*
Pre-migration education	0.34***	0.31***	0.13***	0.17***	0.23***	0.19***	0.50***	0.40***	0.10**	*0.08*	0.07	*0.08*
Post-migration education	0.36***	0.28***	0.19***	0.17***	0.32***	0.25***	0.35***	0.23***	0.18***	0.14***	0.07	*0.05*
Employment status—employed	0.26***	0.12**	0.05	*0.05*	0.19***	0.10*	0.35***	0.11**	0.17***	*0.08*	0.06	*0.07*
Pre-migration work experience	0.14***	* − 0.02*	0.05	* − 0.01*	0.09*	* − 0.01*	0.24***	*0.03*	− 0.01	* − 0.06*	0.02	* − 0.02*
Sub-Saharan African region ^#^	0.19***	0.16***	0.23***	0.22***	0.16***	0.10*	0.11**	0.08*	− 0.07	* − 0.04*	0.21***	0.17***
South Asian region^#^	0.02	*0.01*	− 0.10*	*0.00*	− 0.01	*0.00*	0.11**	*0.00*	0.13***	0.12*	− 0.15***	− 0.18***
East Asia and Pacific^#^	− 0.24***	− 0.14***	− 0.16***	− 0.10**	− 0.22***	− 0.16***	− 0.26***	− 0.15***	0.00	*0.04*	− 0.08*	* − 0.04*
Religion—Muslim^@^	− 0.03	* − 0.02*	0.02	*0.06*	− 0.02	* − 0.06*	− 0.06	* − 0.02*	− 0.02	* − 0.09*	0.01	*0.09*
Religion—other^@^	− 0.05	* − 0.08*	− 0.21***	− 0.13**	− 0.09*	− 0.13**	0.13***	*0.05*	0.11**	*0.02*	− 0.15***	* − 0.07*
Refugee^	− 0.09*	0.11**	0.09*	0.19***	− 0.05	*0.08*	− 0.21***	*0.06*	− 0.12**	* − 0.04*	0.01	*0.02*
*R*^2^		0.32		0.17		0.23		0.44		0.08		0.10
*F*-value		21.80***		9.16***		13.26***		36.29***		4.03***		4.92***

Significance levels: ***p < 0.001, **p < 0.01, *p < 0.05

Checked for multicollinearity and found all VIF values to be below 3 (maximum VIF = 2.11)

^#^Reference group: Middle East and North African region

^@^Reference group for religion: Christian

^Reference is all other migrant types

Overall, findings from bivariate analyses were consistent in the multivariate regressions. In the multivariate regression models, demographic and migration-related factors explained 32% of the variance in SSL. After controlling for factors in the models, pre-migration ( *β* = 0.31; *p* < 0.001) and post-migration (*β* = 0.28; *p* < 0.001) educational attainment, being employed in Australia (*β* = 0.12; *p* < 0.01), and being a refugee (*β* = 0.11; *p* < 0.01) were positively associated with the overall SSL. In contrast, age (*β* =  − 0.10; *p* < 0.001) was negatively associated with the overall SSL. Also, compared to migrants from the Middle East and North Africa (MENA) region, migrants from the sub-Saharan African region had a higher level of SSL (*β* = 0.16; *p* < 0.001), while in contrast, those from the East Asia and Pacific (EAP) region had a lower level of SSL (*β* =  − 0.14; *p* < 0.001). Migrants from South Asia did not differ significantly in their overall SSL from the reference group (MENA). Results did not show support for the association between level of overall SSL and time since migration into Australia, marital status, gender, pre-migration work experience or religion.

### Knowledge

Overall, demographic and migration-related factors explained 17% of the variance in the knowledge component of SL. The multivariate OLS regression results showed that pre-migration education (*β* = 0.17; *p* < 0.001), post-migration education (*β* = 0.17; *p* < 0.001) and being a refugee (*β* = 0.19; *p* < 0.001) were positively associated with the SSL dimension of knowledge. In comparison to migrants who identified as Christians, those who identified their religion as other than Christianity or Islam had a lower level of knowledge (*β* =  − 0.13; *p* < 0.01). Muslims did not differ significantly in their level of knowledge dimension of SSL from the Christian migrants. Similar to results of overall SSL, compared to the reference group of migrants from MENA region, migrants from the sub-Saharan region have a higher level of knowledge dimension of SSL (*β* = 0.22; *p* < 0.001). In contrast, migrants from the East Asia and Pacific region had a lower level of the knowledge dimension of SSL (*β* =  − 0.10; *p* < 0.01) as compared to the reference group of MENA region. Migrants from South Asia did not differ significantly in their level of knowledge from the MENA reference group. Results did not show support for the association between level of knowledge and time since migration into Australia, age, marital status, gender, pre-migration work experience or employment status.

### Empowerment

Overall, demographic and migration-related factors explained 23% of the variance in the empowerment component of SSL. The multivariate OLS regression results showed that pre-migration education (*β* = 0.19; *p* < 0.001), post-migration education (*β* = 0.25; *p* < 0.001), and employment in Australia (*β* = 0.10; *p* < 0.05) were positively associated with the SSL dimension of empowerment. Age of migrants was negatively associated with their level of empowerment (*β* =  − 0.09; *p* < 0.05). In comparison to Christians, migrants who reported their religion as other than Christianity or Islam reported a lower level of empowerment (*β* =  − 0.13; *p* < 0.01). Muslims did not differ significantly in their level of empowerment dimension from Christians. Compared to migrants from the MENA region, level of empowerment was significantly higher among migrants from sub-Saharan Africa (*β* = 0.10; *p* < 0.05) but lower among migrants from the EAP region (*β* =  − 0.16; *p* < 0.001). Migrants from South Asia did not differ significantly in their level of empowerment from the MENA reference group. Results did not show support for the association between level of empowerment and time since migration into Australia, marital status, gender, pre-migration work experience or migrant status.

### Competency

Overall, demographic and migration-related factors explained 44% of the variance in competency component of SSL and this was the highest *r*-squared across the components or overall SSL. The multivariate OLS regression results showed that pre-migration education (*β* = 0.40; *p* < 0.001), post-migration education (*β* = 0.23; *p* < 0.001), and employment in Australia (*β* = 0.11; *p* < 0.01) were positively associated with competency dimension of the SSL. Migrants’ age was negatively associated with their level of competency (*β* =  − 0.22; *p* < 0.001). Compared to migrants from the MENA region, migrants from sub-Saharan Africa scored higher on competency (*β* = 0.08; *p* < 0.05) while those from the EAP region had a lower level of competency (*β* =  − 0.15; *p* < 0.001). Migrants from South Asia did not differ significantly in their level of competency from those from the MENA region. Results did not show support for the association between level of competency and time since migration into Australia, marital status, gender, pre-migration work experience, religion and migration type.

### Community Influence

Overall, demographic and migration-related factors explained 8% of the variance in competency component of SSL, which was the lowest r-squared across the components or overall SSL. Multivariate OLS regression results showed that post-migration education (*β* = 0.14; *p* < 0.001) was positively associated with community influence. Also, compared to migrants from the MENA region, migrants from the South Asia region scored higher on community influence dimension (*β* = 0.12; *p* < 0.05). However, migrants from sub-Saharan Africa and the EAP region did not differ significantly in their level of community influence from those from the MENA region. Results did not show support for the association between community influence and time since migration into Australia, age, marital status, gender, pre-migration education, pre-migration work experience, employment status, religion and migration type.

### Political Component

Overall, demographic and migration-related factors explained 10% of the variance in the political component of SSL. Multivariate OLS regression results showed that time in Australia (*β* = 0.09; *p* < 0.05) was positively associated with the political dimension of SSL. Compared to migrants from the MENA region, migrants from the sub-Saharan Africa scored higher on the political dimension (*β* = 0.17; *p* < 0.001) while those from the South Asia region scored lower (*β* =  − 0.18; *p* < 0.001). Migrants from the EAP region did not differ significantly from those from the MENA reference group. Results did not show support for the association between the political dimension and age, marital status, gender, pre- or post-migration educational attainment, employment status, pre-migration work experience, religion and migration type.

### Analysis of Demographic and Migration-related Factors Across SSL Dimensions

#### Education

Post-migration education appeared to be the most significant positive factor and was the only factor associated with all but the political SSL dimension as well as the overall SSL, with standardized *β* coefficients ranging between 0.05 (*p* > 0.05) for the political dimension to 0.25 (*p* < 0.001) for empowerment. Pre-migration education was also positively associated with most dimensions, except the community and political dimensions, with standardized *β* coefficients ranging from 0.08 (*p* > 0.05) to 0.40 (*p* < 0.001).

#### Employment Status

Employment status in Australia was positively associated with competency (*β* = 0.11, *p* < 0.01) and empowerment (*β* = 0.10,* p* < 0.05) dimensions, but not others. Pre-migration employment did not statistically impact on SSL or any of its components.

#### Region of Origin

Compared to migrants from the reference group of MENA region, migrants from the sub-Saharan Africa scored comparatively higher on all dimensions of SSL, except community influence, with the highest comparative score on knowledge (*β* = 0.22, *p* < 0.001) while comparative scores for migrants from the EAP region were significantly lower on most dimensions of SSL (knowledge, empowerment, and competency), with a lowest comparative score on empowerment (*β* =  − 0.16, *p* < 0.001). Interestingly, migrants from the South Asian region scored higher than the comparative group of MENA on community influence (*β* = 0.12,* p* < 0.05), but lower than those from the MENA on the political dimension (*β* =  − 0.18,* p* < 0.001).

#### Religion

Compared to Christians, those with a religion other than Christianity or Islam scored significantly lower on knowledge and empowerment (*β* =  − 0.13, *p* < 0.01). Results did not show support for significant differences among religious groups of on any other SSL dimensions.

#### Migrant Type

Compared to all other migrant types, refugees scored significantly higher on knowledge (*β* = 0.19, *p* < 0.001). Results did not show support for significant differences among types of migrants on any other SSL dimensions.

#### Age

Age was negatively associated with only empowerment (*β* =  − 0.09, *p* < 0.05) and competency (*β* =  *− *0.22, *p* < 0.001), and insignificant for all other SSL dimensions.

#### Gender

There were no significant differences across SSL dimensions between males and females.

#### Time in Australia

Time in Australia was positively associated with the political dimension, only (*β* = 0.09, *p* < 0.05), but not associated with any other SSL dimensions.

Marital status did not appear to be significantly associated with any of the dimensions of SSL or overall SSL.

## Discussion

At the higher level of analysis, many of the demographic and migration-related factors examined (i.e. 7 of the 9 explored) are important for overall SSL or components of SSL. Of these 9 factors, only gender and marital status did not appear to be significantly associated with any of the dimensions of SSL, whilst only years lived in Australia (Political) impacted on one SSL factor, whereas the other 6 factors impacted on at least two components of SSL and/or overall SSL. Apart from age that showed negative associations, six factors were positively associated with SSL dimensions. However, for religion and region of origin, there were mixed trends with negative effects depicted for religion other than Christianity or Islam (knowledge and empowerment) and the EAP region (knowledge, empowerment and competency) and south Asia (political) as regions of origin. These results point to the complexity of migratory pathways.

The results are important as they seem to suggest that Australia theoretically could improve settlement outcomes (i.e. higher SSL) by selecting migrants with certain characteristics, that is, younger migrants and those with higher levels of pre-migration education. Whether this is a realistic option within the UNCHR process is not clear. From an ideological perspective, this would not be ideal as all refugees need to be supported, regardless of difficulty engaging in their new host country. The UNHCR resettlement submissions are based on the three priority levels: emergency priorities (i.e. cases in which the immediacy of security and/or medical condition necessitates removal from the threatening conditions within a few days, if not within hours), urgent priorities (e.g. refugees have serious medical risks or other vulnerabilities requiring expedited resettlement within 6 weeks of submission) and normal (e.g. no immediate medical, social or security concerns which would merit expedited processing) (United Nations High Commissioner for Refugees, 2011). Nevertheless, our findings suggest that suggest that settlement and integration policies in Australia need to be informed by migrants’ pre-migration age and educational attainment, but not pre-migration employment experiences. Our data contradict findings by Udah and colleagues (Udah et al., 2019) who contend that Australian settlement and integration policies need to be informed by migrants’ employment experiences.

Our findings illustrate that those admitted under the refugees and humanitarian entrants’ scheme scored higher on the overall SSL and the knowledge dimension are encouraging given pre- and post-arrival orientation programs. The pre-migration Australian Cultural Orientation program targets only refugees and humanitarian entrants to help them master and deal with challenges associated with travelling to Australia (the journey) and settling in Australia including on-arrival assistance and settlement services they have access to and the right to use (health care such as Medicare, hospitals, immunisation programs and preventative health; education programs including English lessons education pathways for children and parents; employment including welfare entitlements through Centrelink, recognition of overseas skills and qualifications and job-searching services; financial literacy including money management, banking, budgeting and taxation; housing options; transport requirements and Australian laws). The pre-arrival orientation program is complemented with the post-arrival orientation provided as part of settlement programs such as the Adult Migrant English Program, the HSP, the SETS and the Skills for Education and Employment program (Commonwealth of Australia, 2021; Department of Home Affairs, 2020a, b).

Our findings that sub-Saharan African migrants have higher overall SSL and all SSL dimension (except community influence) than migrants from other regions contradicts the political narrative that has been postulated over the last 20 years, contending that migrants from this region fail to settle in Australia due to their low level of literacy and associated poor settlement outcomes (Johnson, 2018; Koziol & Cunningham, 2018; Pearlman, 2007; Udah et al., 2019). Such narrative suggests that sub-Saharan migrants are disproportionately over-represented in gang groups, mis-use and abuse alcohol, fighting at nightclubs and family infighting (Johnson, 2018; Koziol & Cunningham, 2018; Pearlman, 2007; Udah et al., 2019). These narratives are often grounded in stereotypes and racial assumptions and are not supported by the data. Our findings are consistent with data-based evidence (Renzaho et al., 2022a).

The results highlight the ability to identify migrants who will need additional support to assist in better integrated across component of SSL or the overall SSL. Those who are older appear to have more difficulties, as do those of ethnic EAP and other religions. Interestingly, South Asian migrants need additional support in one area (political dimension) but have higher SSL outcomes in another area (community influence). Thus, developing targeted programs is essential. The results that being in Australia will implicitly assist individuals is enhancing SSL seems to be disproven in this study, as the only instance where being in Australia longer appears to benefit migrants is in the political domain. In a desire to create more opportunities for migrants, there is often a policy focus on employment and education. Those working in Australia appears to only improve overall SSL as well as empowerment and competence components of SSL. It is, perhaps, surprising that this did not impact on all components, whereas post-migration education had a greater positive impact across overall SSL and all components other than the political component. These results do suggest that jobs in Australia and education are keys for integration, but more emphasis should be on post-migration education.

Finally, it has frequently been postulated that female migrants, especially refugees, are disadvantaged and have trouble integrating into Australia and other OECD countries (Australian Human Rights Commission, 2013; Desiderio, 2020; Liebig & Tronstad, 2018). However, the results of this study seem to suggest that there is no difference in terms of the impact on SSL. This of course may simply mean that both sexes have difficulties across the range of areas. Additionally, if we were to explore interactions between demographic factors, we may still find that in some communities female members face more challenges engaging in the Australian society and economy (Australian Human Rights Commission, 2013; Desiderio, 2020; Liebig & Tronstad, 2018). It may be also that different usages of integration and settlement measures may explain differences in outcomes between studies.

Our study has some limitations worth outlining. It was cross-sectional in nature and hence the results are correlations and causality cannot be implied. Australia has funded a longitudinal study—the “Building a New Life in Australia” which started in 2013 and has been documenting how humanitarian migrants settle into life in Australia. Integrating the SSL components may elucidate the directions of the associations between SSL dimensions and migrants’ demographic and migration-related factors. The use of snowballing technique means that the representativeness of the sample cannot be ascertained with accuracy. This is because the sampling bias may closely associate with initial subjects nominating people whom they know well and are similar to them, and with shared traits and characteristics. While snowball recruitment is frequently used to access hard-to-reach communities (Leighton et al., 2021), it can have limitations where respondents only suggest others like themselves. We did seek to limit this by restricting people in family networks to get a broader cross section of migrants.

## Conclusion

Our research highlights the need for further research not simply on SSL, but on whether different types of SSL lead to better integration and settlement outcomes. We identified that demographic characteristics impact on SSL; however, it may be the case that different levels of SSL also result in better inclusion, sociocultural and economic outcomes. Future research needs to explore the relationships between SSL, settlement outcomes and other factors. Overall, the results do appear to suggest that governments can use these results to design programs targeting specific SSL needs for different communities, and thus potentially developed targeted adaptations to programs surviving given communities or types of migrants. The present approach by governments is a ‘one size fits all’ settlement services and this research suggests that, based on the refugees and humanitarian entrants’ backgrounds and demographics, they may in fact need more targeted programs. There may be a broader need to not only to deliver programs, but better align the programs with specific objectives. This is especially important for new migrants who do not know. what they do not know about the Australian socio-economic environment. Thus, individuals need to also understand how new environments differ to their home country environments.

Integrating the supports into a broader sense of inclusion and explaining how these programs can assist in social and economic integration is, therefore, an important addition to simply delivering programs.

While an initial reaction may be that these adaptions and extensions will be too costly to have more targeted actions, in fact it would mean that some program funding could potentially be saved, by not targeting groups who already have these specific skills and abilities, in regard to settlement services. Enhanced programs also would improve outcomes and resulting in a higher return on social investment. Given the OECD identified that each migrant provides significant benefits to their host countries, these might better be seen as investments in future growth rather than simply be seen as a financial expenditure. Thus, the ways in which policy makers view services may need to also evolve thereby potentially shaping overall system improvements as well as fostering more social and economic inclusion. Therefore, the results appear to suggest that it may be possible to realign existing programs more effectively rather than designing new programs, with targeted actions being more cost effective (i.e., targeting those in need), thereby creating the ideal win–win outcome of more effective implementation and better social/economic integration.

## Data Availability

The authors confirm that the data supporting the findings of this study are available and have been provided as supplementary materials.
